# Identifying anatomical subtypes of sporadic EOAD in LEADS via unsupervised clustering of MRI‐based regional atrophy patterns

**DOI:** 10.1002/alz.093106

**Published:** 2025-01-09

**Authors:** Scott M McGinnis, Yuta Katsumi, Ryan Eckbo, Michael Brickhouse, Ani Eloyan, Kelly N. Nudelman, Tatiana M. Foroud, Jeffrey L. Dage, Maria C. Carrillo, Gil D. Rabinovici, Liana G. Apostolova, Alexandra Touroutoglou, Bradford C. Dickerson

**Affiliations:** ^1^ Frontotemporal Disorders Unit and Massachusetts Alzheimer's Disease Research Center, Department of Neurology, Massachusetts General Hospital and Harvard Medical School, Boston, MA USA; ^2^ Frontotemporal Disorders Unit, Department of Neurology, Massachusetts General Hospital and Harvard Medical School, Boston, MA USA; ^3^ Frontotemporal Disorders Unit and Massachusetts Alzheimer’s Disease Research Center, Department of Neurology, Massachusetts General Hospital and Harvard Medical School, Boston, MA USA; ^4^ Massachusetts General Hospital and Harvard Medical School, Boston, MA USA; ^5^ Department of Biostatistics, Brown University, Providence, RI USA; ^6^ Department of Medical and Molecular Genetics, Indiana University School of Medicine, Indianapolis, IN USA; ^7^ Department of Neurology, Indiana School of Medicine, Indianapolis, IN USA; ^8^ Alzheimer's Association, Chicago, IL USA; ^9^ Department of Neurology, Memory and Aging Center, University of California San Francisco, San Francisco, CA USA; ^10^ Department of Radiology and Imaging Sciences, Indiana University School of Medicine, Indianapolis, IN USA; ^11^ Department of Neurology, Indiana University School of Medicine, Indianapolis, IN USA

## Abstract

**Background:**

Neurodegeneration in sporadic early‐onset Alzheimer disease (EOAD) is topographically heterogeneous, as suggested by variability in syndromic presentation. We performed an unsupervised clustering analysis of structural MRI data to identify anatomical subtypes of EOAD. We hypothesized that distinct clusters will be present but will: (1) share areas of overlap focused around posterior regions of our newly developed EOAD signature of cortical atrophy (Touroutoglou et al., 2023), including the posterior default mode (DMN) and frontoparietal control networks (FPN) of the cerebral cortex; and (2) show non‐overlapping topography inclusive of nodes of other networks including dorsal attention (DAN) and visual association (VIS) networks.

**Methods:**

We analyzed structural MRI data from 183 individuals with EOAD and 88 cognitively unimpaired (CU) participants from the Longitudinal Early‐Onset Alzheimer's Disease Study (LEADS). MRI data were processed using FreeSurfer v6.0 to estimate vertex‐wise cortical thickness, which was converted to W‐scores (i.e., Z‐scores relative to CU participants adjusted for age and sex). We then performed an agglomerative hierarchical clustering analysis on a between‐patients similarity matrix computed from rank‐ordered whole‐cortex W‐scores.

**Results:**

Analysis yielded 2 major clusters, with subordinate clustering failing to delineate additional unique topographies. One cluster (n=54) exhibited prominent atrophy in the anterior DMN (medial prefrontal cortex, anterior lateral temporal cortex) and rostral FPN (rostral middle and superior frontal gyri). The other cluster (n=129) showed prominent atrophy in the DAN (superior parietal lobule, caudal superior frontal gyrus, posterior temporal cortex) and VIS (posterior inferior temporal/occipital cortex, posterior parietal cortex). Both clusters showed atrophy in the posterior DMN (posterior cingulate cortex, precuneus, posterior inferior parietal lobule, mid lateral temporal cortex) and the FPN (middle and superior frontal gyri, anterior inferior parietal lobule, mid inferior temporal cortex). The clusters did not differ with respect to age, sex, education, APOE status, or clinical measures of disease severity.

**Conclusions:**

Our sample of sporadic EOAD patients comprised 2 principal anatomical subtypes, commonly overlapping with the posterior DMN and FPN that constitute the EOAD signature, one subtype uniquely overlapped with the anterior DMN/rostral FPN and the other with the DAN/VIS network. Anatomical differences between the subtypes likely correspond to aspects of phenotypic heterogeneity.

